# Beyond the Eyes: Clinico-Radiological Correlation of Bilateral Complete Horizontal Gaze Palsy in Moebius Syndrome

**DOI:** 10.7759/cureus.60887

**Published:** 2024-05-23

**Authors:** Alamelu Alagappan, Biswamohan Mishra, Biswajit Sahoo, Suprava Naik, Manoj K Nayak

**Affiliations:** 1 Radiodiagnosis, All India Institute of Medical Sciences, Bhubaneswar, Bhubaneswar, IND; 2 Neurology, All India Institute of Medical Sciences, New Delhi, New Delhi, IND

**Keywords:** facial collicular hypoplasia, bilateral abducens palsy, facial palsy, bilateral complete horizontal gaze palsy, moebius syndrome

## Abstract

Moebius syndrome is a rare disease characterized by unilateral or bilateral facial nerve palsies with/without other cranial nerve palsy. It manifests clinically with facial muscle weakness and/or ophthalmoplegia and can be associated with other physical anomalies such as various limb deformities and orofacial malformation. Herein, we have described the clinical and radiological features of Moebius syndrome in a 9-year-old female child who presented with left-side facial palsy and bilateral complete horizontal gaze palsy.

## Introduction

Moebius syndrome is a rare disease with unilateral or bilateral facial nerve palsies with/without other cranial nerve palsy. It manifests clinically with facial muscle weakness and/or external ophthalmoplegia. With an estimated incidence of about two to 20 per million live births, Moebius syndrome has been documented in the literature in only 300 cases so far [1]. Horizontal gaze palsy is a common ocular manifestation due to pontine abducens nuclear defects [2,3]. Due to the absence of bilateral facial colliculi, there will be the fourth ventricular floor flattening, the most common neuroradiological finding in this syndrome [2]. The diagnosis of Moebius syndrome is primarily based on the clinical features primarily caused by muscular abnormalities resulting from nerve defects. Although there isn’t a specific test to diagnose the syndrome, an MRI of the brain confirms the diagnosis of Moebius syndrome, demonstrates imaging abnormalities of the brain, and excludes mimicker of Moebius syndrome. Herein, we have described a case of Moebius syndrome with bilateral horizontal gaze palsy, left side facial palsy, with characteristic radiological features in a nine-year-old female child.

## Case presentation

**Figure 1:**
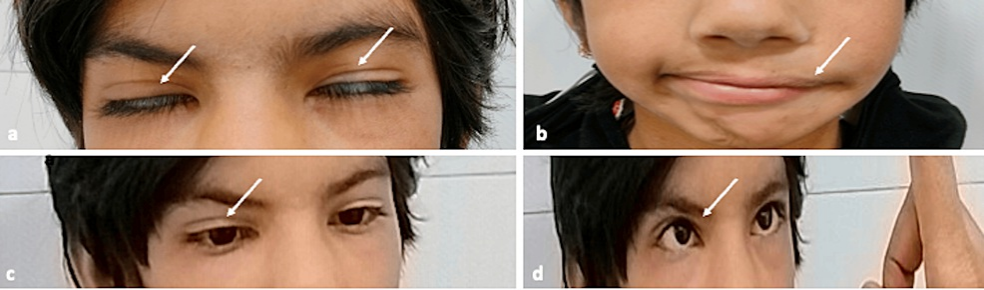
Clinical images showing the facial and ophthalmologic features. Clinical photograph showing incomplete closure of both eyelids (arrows in (a)) and facial palsy (arrow in (b)). Clinical picture showing the loss of abduction in the right eye (arrow in (c)) with normal convergence (arrow in (d)).

A 9-year-old female presented with symptoms of inability to close her left eye since birth. Her parents gave a history of an asymmetrical smile, drooling of saliva from the left side and incomplete left eye closure since birth. She was born as a full-term neonate with normal vaginal delivery and cried immediately after birth. However, the child had no abnormal developmental history since birth. There was no previous history of use of misoprostol. On clinical examination, there were asymmetric facial features with a right-sided deviation of the angle of the mouth, lack of prominent left nasolabial fold, and difficulty in the closure of the left eye completely. Other features were bilateral horizontal gaze palsy with total restriction on abduction and conjugate adduction of the contralateral eye with normal convergence, possibly due to involvement of the left side facial nerve and bilateral abducens nerve (Figure 1a-1d).

**Figure 2:**
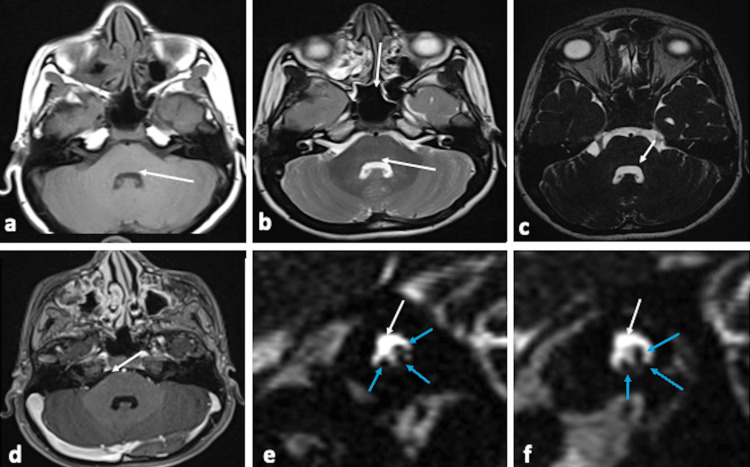
Magnetic resonance imaging (MRI) images showed the imaging features of Moebius syndrome. MRI Axial T1 (a), T2 (b), and FIESTA (fast imaging employing steady-state acquisition) (c) images showed flattening of the floor of the fourth ventricle with the absence of bilateral facial colliculus (white arrows), and post-contrast T1 (d) image showed absent cisternal segments of bilateral abducens nerves (white arrow). Sagittal MRI image (FIESTA sequences) (e,f) showed the absence of the facial nerves (white arrows in (e,f)) with intact superior, inferior vestibular, and cochlear nerves (blue arrows in (e,f)) within both internal auditory canals.

Neurological examination showed left facial nerve palsy and bilateral abducens nerve palsy with bilateral complete horizontal palsy. Reflexes, tone and joint position sense were normal bilaterally. No other orofacial or limb abnormalities, tongue atrophy, or signs of other cranial nerve palsies were noted. The brain's magnetic resonance imaging (MRI) showed flattening of the fourth ventricular floor, with a lack of prominence of bilateral facial colliculi, absent bilateral facial nerves in the internal auditory canals, and absent bilateral abducent nerves cisternal segments (Figure 2a-2f). 

After the MRI, on careful clinical evaluation, there was a mild suspicion of weakness in the right eyebrow movements. A diagnosis of Moebius syndrome for this patient was made based on these clinical and radiological findings. Subsequently, she was transferred to ophthalmology for ocular care and further management.

## Discussion

Moebius syndrome is a clinical condition initially described to have bilateral simultaneous abducent and facial cranial nerve palsies. It was first described by Paul Julius Moebius in 1892. However, recently, it has been defined as a non-progressive congenital total or partial, unilateral or bilateral facial nerve palsy and physical anomalies such as various limb deformities and orofacial malformation (talipes, micrognathia, congenital absence of muscles, especially the pectoral group).

Different hypotheses were postulated like genetically based developmental rhombomeric abnormality that affects the facial cranial nerve nuclei, or a disruption in the brainstem’s vascular supply as a result of external, mechanical, or genetic abnormalities that causes ischemia of the facial nerve nuclei region or intake of drugs including thalidomide, misoprostol, and cocaine during antenatal period [3]. 

Verzijl et al. recently reported the primary criteria, including facial nerve palsy with ocular abduction impairment, with/without association with additional cranial nerve palsies, orofacial issues, or musculoskeletal abnormalities [4]. Although our case has no limb or orofacial deformities, it meets the primary criteria for Moebius syndrome. This reported case had bilateral horizontal gaze palsy (total restriction of bilateral abduction and conjugate adduction of the contralateral eye). A similar case report was presented by Kulkarni et al., which included bilateral lateral rectus palsy, partial medial rectus palsy, and partial restriction of convergence [5]. Convergence was intact in our case, indicating the possibility of equal dysgenesis bilateral abducent nucleus motor neurons and interneurons of abducent nucleus destined to median longitudinal fasciculus. However, a smooth pursuit of vertical gaze with a full range of movements was noted.

Rucker et al. reported that phenotypically distinct ocular motor findings can be identified in these patients, like bilateral horizontal gaze palsy with vertical range impairments, impaired abduction, full ocular motor range, and isolated bilateral horizontal gaze palsy. Bilateral horizontal gaze palsy was the most common ocular phenotype observed in 43% of the patients [1]. Our case also had normal convergence with bilateral horizontal gaze palsy, corresponding to bilateral abducent nuclear involvement rather than the sixth nerve involvement. Our patient clinically had left facial palsy; however, radiologically, there is a discrepancy between the lack of prominence of bilateral facial colliculi and the absence of bilateral facial nerves in the internal auditory canals suggestive of bilateral facial nerve involvement.

Falco et al., in their article, reported that these clinically varied presentations could be due to traumatic aetiology. But in our case, the traumatic aetiology can be disregarded as there is bilateral abducent nerve involvement causing bilateral horizontal gaze palsy, bilateral absence of facial colliculi on MRI, absent facial nerves in the internal auditory canal, and the patient had no history of birth trauma or forceps delivery [6]. We suggest that this clinical discrepancy can be due to the fact of the complete expression of left facial palsy features (grade IV - moderately severe dysfunction) masking the underlying mild right facial palsy features.

On careful evaluation, a slight suspicious weakness in right eyebrow movements was observed, belonging to grade II (mild dysfunction) House-Brackmann grading on the right side. However, the varied presentations of facial palsy are still unexplained in the literature. MRI demonstrates the cranial nerve and brainstem nuclei abnormalities of this syndrome. Radiologic features can be the flattening of the floor of the fourth ventricle with the absence of bilateral facial colliculi, pontine hypoplasia, increased anteroposterior diameter of the midbrain, tectal beaking, dysplasia of corpus callosum, ventriculomegaly and fusion of thalami [3]. 

The diagnosis of Moebius syndrome primarily depends upon the symptoms and clinical features; however, brain imaging can significantly describe the brain's features and help exclude other similar pathologies [7]. The differential diagnosis can be Melkerrson-Rosenthal syndrome, Poland syndrome, hereditary congenital facial palsy, Duane retraction syndrome, congenital fibrosis of the extraocular muscles, DiGeorge syndrome, and CHARGE syndrome [7]. Melkerrson-Rosenthal syndrome can have a triad of tongue fissures, lip swelling, and congenital facial palsy, and Poland syndrome can have ipsilateral pectoralis muscle hypoplasia with congenital palsy of abducens and facial nerves. Hereditary congenital facial palsy is characterized by isolated facial nerve palsy without abducens nerve involvement, and Duane retraction syndrome can have a retraction of eyeballs with limited abduction or adduction movements and shortening of palpebral fissure on horizontal eye movements [7]. 

Congenital fibrosis of the extraocular muscles can cause vertical gaze palsy and ptosis with severe congenital strabismus. Since the disease is congenital and nonprogressive, no definitive treatment is described. Hence, only symptomatic treatment can be given. The morbidity of this disease can be reduced through various interventions, including proper ocular care through procedures such as tarsorrhaphy, strict monitoring of potential complications, patient education, and a collaborative approach involving multiple medical professionals [8]. The prognosis of the child may be improved by early intervention that incorporates visual and sensory information with a focus on emotional issues.

## Conclusions

This case report emphasizes the necessity of early recognition and diagnosis of Moebius syndrome in children, as it can significantly impact their overall prognosis and quality of life. The most common presentation to the clinic is facial palsy. Bilateral complete horizontal gaze palsy is the most common ocular presentation. Though the radiological findings aren’t part of the diagnostic criteria, they play a significant role in aiding the diagnosis. There is currently no specific treatment for Moebius patients. As such, more and more such cases need to be reported to build a large cohort, which can be used to conduct definitive therapeutic trials in the future.

## References

[1] Rucker JC, Webb BD, Frempong T, Gaspar H, Naidich TP, Jabs EW (2014). Characterization of ocular motor deficits in congenital facial weakness: Moebius and related syndromes. Brain.

[2] Herrera DA, Ruge NO, Florez MM, Vargas SA, Ochoa-Escudero M, Castillo M (2019). Neuroimaging findings in Moebius sequence. AJNR Am J Neuroradiol.

[3] Bavinck JN, Weaver DD (1986). Subclavian artery supply disruption sequence: hypothesis of a vascular etiology for Poland, Klippel-Feil, and Möbius anomalies. Am J Med Genet.

[4] Verzijl HT, van der Zwaag B, Cruysberg JR, Padberg GW (2003). Möbius syndrome redefined: a syndrome of rhombencephalic maldevelopment. Neurology.

[5] Kulkarni A, Madhavi MR, Nagasudha M, Bhavi S (2012). A rare case of Moebius sequence. Indian J Ophthalmol.

[6] Falco NA, Eriksson E (1990). Facial nerve palsy in the newborn: incidence and outcome. Plast Reconstr Surg.

[7] Zaidi SM, Syed IN, Tahir U, Noor T, Choudhry MS (2023). Moebius syndrome: what we know so far. Cureus.

[8] Pedersen LK, Maimburg RD, Hertz JM, Gjørup H, Pedersen TK, Møller-Madsen B, Østergaard JR (2017). Moebius sequence -a multidisciplinary clinical approach. Orphanet J Rare Dis.

